# The Enigma That Is ROHHAD Syndrome: Challenges and Future Strategies

**DOI:** 10.3390/brainsci14111046

**Published:** 2024-10-23

**Authors:** Katherine Hawton, Dinesh Giri, Elizabeth Crowne, Rosemary Greenwood, Julian Hamilton-Shield

**Affiliations:** 1 Bristol Royal Hospital for Children, University Hospitals Bristol and Weston NHS Foundation Trust, Bristol BS2 8BJ, UK; 2 Translational Health Sciences, University of Bristol, Bristol BS8 1QU, UK; 3 York Trials Unit, Health Sciences, University of York, York YO10 5DD, UK; 4 NIHR Biomedical Research Centre (Diet and Physical Activity Theme), University of Bristol, Bristol BS2 8BJ, UK

**Keywords:** ROHHAD, obesity, hypoventilation, hypothalamic, autonomic

## Abstract

Rapid-onset obesity with hypoventilation, hypothalamic dysfunction, and autonomic dysregulation (ROHHAD) is a rare syndrome presenting in early childhood associated with a high risk of mortality between 50 and 60%. It is characterised by rapid, early onset of obesity between 1.5–7 years, along with central hypoventilation and hypothalamic dysfunction, such as central hypothyroidism, hyperprolactinemia, disorders of sodium and water balance, growth hormone deficiency, adrenocortical insufficiency, or disorders of puberty and features of autonomic dysregulation. Up to half of cases have neural crest tumours, most commonly ganglioneuromas or ganglioneuroblastomas. The incidence of ROHHAD syndrome in any population is unknown. Currently, there is no specific diagnostic or genetic biomarker for ROHHAD, and diagnosis is based on clinical signs and symptoms, which is often challenging, and consequently may be delayed or unrecognised. Early diagnosis is important, as without intervention, ROHHAD is associated with high morbidity and mortality. Aetiology remains unclear; an autoimmune origin has been postulated, with immunosuppressive agents being used with variable benefit. With no cure, multidisciplinary management is largely supportive. Therefore, there are many unanswered questions in ROHHAD syndrome. In this review article, we outline the challenges posed by ROHHAD syndrome, including aetiology, genetics, diagnosis, screening, management, and prognosis. We present research priorities to tackle these issues to improve outcomes.

## 1. Introduction

Rapid-onset obesity with hypoventilation, hypothalamic dysfunction, and autonomic dysregulation (ROHHAD) is a rare syndrome associated with high rates of morbidity and mortality [1]. It usually presents between 1.5 and 7 years of age with rapid onset of obesity and subsequently central hypoventilation, hypothalamic dysfunction, and autonomic dysregulation. It was initially described as primary alveolar hypoventilation syndrome in 1965 [2] and subsequently late-onset central hypoventilation with hypothalamic dysfunction (LO-CHS/HD) [3]. The name ROHHAD was introduced along with proposed diagnostic criteria in 2007 [4]. The acronym was expanded to ROHHAD-NET to encompass the risk of neuro-endocrine tumours (NET), such as ganglioneuroma or ganglioneuroblastomas, which are reported in up to half of patients with ROHHAD [5]. 

Clinical features of ROHHAD are largely agreed to include rapid onset of obesity, central hypoventilation, and any features of hypothalamic dysfunction with or without autonomic dysfunction [4], with each feature often, although not always, emerging in the order of the acronym [6]. Features of hypothalamic dysfunction may include hyperprolactinaemia, central hypothyroidism, disordered water and sodium balance, growth hormone deficiency, adrenocorticotropin hormone deficiency, and delayed or precocious puberty. 

Aetiology is unknown, although a variety of genetic, epigenetic, immunological, and neuro-anatomical mechanisms have been proposed. Diagnosis is often challenging and delayed. Management largely comprises symptomatic management of the different clinical features [7,8], such as ventilatory support or hormone replacement therapy. 

A literature review was performed for all articles including the terms “ROHHAD”, “ROHHAD-NET”, “rapid-onset obesity, hypothalamic dysfunction, hypoventilation and autonomic dysregulation”, or “late-onset central hypoventilation” by searching OVID AMED, Embase, Medline, and Emcare databases with no date limits. In this review, the current available evidence regarding incidence, clinical features, aetiology, diagnosis, management, and prognosis in ROHHAD syndrome is presented, thereby identifying aspects still provoking challenge and uncertainty.

## 2. Incidence and Prevalence

The prevalence of ROHHAD is currently unknown and to date there have been no published studies estimating incidence. There have been approximately 200 cases reported worldwide [7,9]. It appears there may be a female preponderance, with one review reporting a female to male ratio of 3.6:1 [1] and another 1.9:1 [6]. There is no clear geographical distribution of the condition. Due to its rarity, limited awareness of the condition and lack of a diagnostic test, it is likely that a proportion of cases go unrecognised [7,10]. 

There have been several incomplete cases reported without the full constellation of clinical features [11], which may represent evolving cases. ROHHAD may also mimic several other aetiologies underlying obesity, including monogenic causes, or endocrine disorders [12,13,14,15]. The complexities of diagnosing the syndrome without the utility of a specific test may restrict cases being diagnosed, and lead to an underestimation of prevalence [1]. Raising awareness of the syndrome and its clinical presentations will improve timely diagnosis, but ultimately the discovery of a diagnostic test would aid estimates of prevalence. High-quality studies to ascertain incidence and prevalence would be beneficial when planning the development of dedicated services to provide care for individuals living with ROHHAD syndrome. 

## 3. Aetiology

The aetiology of ROHHAD syndrome remains unresolved, despite investigation into various potential causes, making targeted treatment challenging. Potential postulated aetiologies include genetic, epigenetic, autoimmune, and environmental factors, and it is possible that there is more than one causative factor in this syndrome. 

### 3.1. Genetic Aetiology

It has been proposed that the syndrome may have a genetic aetiology, particularly given its similarity to several other genetic or syndromic conditions [7], such as Prader-Willi syndrome [16,17], Smith–Magenis syndrome [18], and congenital central hypoventilation syndrome (CCHS) [17]. However, no genetic basis has been found despite extensive investigations of potential candidate genes and looking for a novel genetic cause from both exome and genome sequencing. 

Candidate gene studies have been performed examining various genes associated with a number of similar conditions. Variants in the *PHOX2B* gene are associated with CCHS; however, no association with ROHHAD syndrome has been found [4,19,20,21], and the presence of *PHOX2B* variants is now considered to exclude the diagnosis [4]. 

The brain-derived neurotrophic factor (BDNF)/tyrosine receptor kinase-B (TRKB) pathway has been considered as potentially relevant [4] as it is related to respiratory control [22], the lung-brain axis [23], and the development of obesity in animal models [24]. TRKB is encoded for by neurotrophic receptor tyrosine kinase 2 (*NTRK2*), and neither variants in *BDNF* nor *NTRK2* have been found in individuals with ROHHAD syndrome [4,25]. Although no variants in the *BDNF* gene have been found, it is possible that BDNF insufficiency could contribute to the pathophysiology of this condition, as many features are shared with conditions involving BDNF haploinsufficiency [25]. 

Genes involved in the development of the hypothalamus and autonomic nervous system, including 5-hydroxytryptamine receptor 1 (*HTR1A*), orthopedia (*OTP*), and pituitary adenylate cyclase activating polypeptide (*PACAP*), have also been considered to be possible candidate genes, but no variants were found in a cohort of 25 patients with the syndrome [26]. Additionally, the DNA of seven patients with ROHHAD syndrome was analysed to look for variants in 11 genes implicated in PWS; however, no pathogenic variants were found [16]. 

Whole exome sequencing has been performed in a number of patients, but as of yet no significant variants or copy number variations have been identified [6,27,28]. Furthermore, whole genome sequencing has been performed without identifying any consistent candidate genes [29,30]. It is possible that ROHHAD syndrome may not be a mendelian disorder and is rather driven by other aetiological factors. However, the application of more powerful techniques, such as the utilisation of Bayesian tools for association analyses comparing with whole genome data sets, may be successful in yielding a causal association, as has been the case for other rare diseases [31]. 

### 3.2. Epigenetic Aetiology

An alternative possibility is that the development of the condition is related to epigenetic mechanisms, whereby there are alterations in gene expression or cellular phenotype, without an alteration in the gene nucleotide sequence itself [32]. Epigenetic mechanisms have been identified in the aetiology of other endocrine disorders, such as Beckwith-Wiedemann syndrome (BWS) [7,33]. The report of a discordant presentation in monozygotic twins [34], who were not found to have any coding differences [27], is suggestive of a possible epigenetic phenomenon [8]. The role of epigenetics in the development of the syndrome therefore warrants further exploration. 

### 3.3. Autoimmune Aetiology

It has been proposed that ROHHAD syndrome may be autoimmune in origin, supported by some benefit having been derived in response to immunomodulatory therapy [6,35,36]. The presence of intrathecal oligoclonal bands has been reported in the CSF of patients with the syndrome, suggesting a pathogenic mechanism similar to other immune-mediated central nervous system disorders [37,38,39].

Patients have also been shown to have altered blood lymphocyte subsets and pro-inflammatory cytokine profiles when compared to patients living with obesity alone, further supporting an autoimmune basis for the condition [40]. The molecular characterisation of adipose-tissue-derived cells from one patient demonstrated up- and down-regulation of a number of genes related to immunoregulation and obesity, further supporting the possibility of an auto-immune basis [41].

Anti-pituitary and anti-hypothalamus antibodies have been identified in a patient in association with a transient response to immunosuppression. More recently, the presence of anti-Zinc finger and SCAN domain containing 1 (ZSCAN1) antibodies has been reported in ROHHAD, both in patients with NETs [42,43] and in patients without tumours [43,44,45]. ZSCAN1 is a protein involved in transcriptional regulation found in the human nervous system, testis, breast, lung, and heart, with RNA expression concentrated in the pituitary gland and hypothalamus, and other areas that may be related to the ROHHAD phenotype [7,46]. The sensitivity and specificity of anti-ZSCAN1 antibodies in this syndrome need to be established, and further evaluation is needed to assess the clinical utility of this as a diagnostic test or therapeutic target. 

### 3.4. Neuro-Anatomical Aetiology

Neuro-anatomical pathology has also been explored as an underlying cause for the syndrome, but there have not been any consistent findings [8,14,47]. There have been reports of magnetic resonance imaging (MRI) demonstrating focal inflammation in the periaqueductal grey matter and hypothalamus [48,49] and hypo-intensities in the pons and midbrain [4]. Hypothalamic inflammation has been found on histopathology in two patients, but this was accompanied by NETs [50,51]. Cerebral atrophy has also been described on both CT and MRI, in association with normal pituitary imaging [8,52]. Hypothalamic inflammation has been associated with more severe behavioural manifestations [8,47,50,51]. If ROHHAD was solely related to an auto-inflammatory process and subsequent neuronal loss in brain regions, it would be expected that imaging findings and histopathological examination of the brain would be more consistent [7].

## 4. Clinical Features

**Figure 1:**
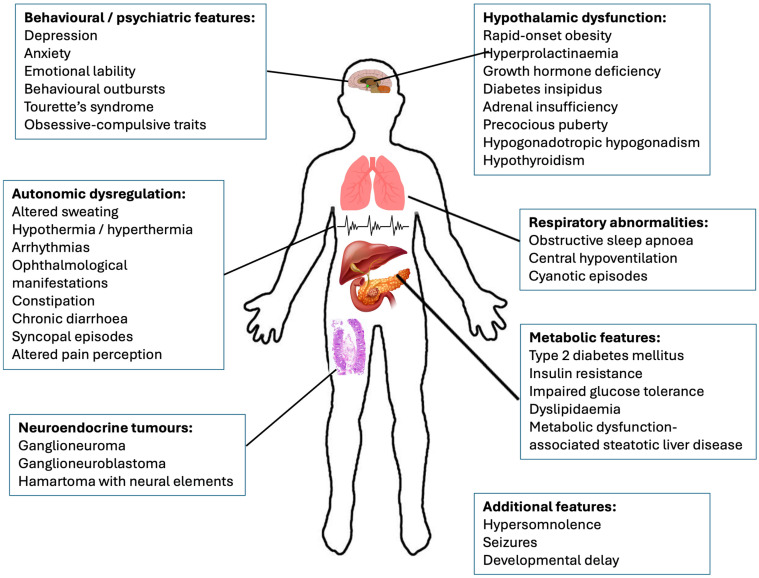
Clinical features in ROHHAD syndrome.

ROHHAD syndrome consists of rapid-onset obesity, hypoventilation, hypothalamic dysfunction, and autonomic dysregulation [4] and neuroendocrine tumours (ROHHAD-NET) [4]. The rapid onset of obesity generally occurs between the ages of 1.5 and 7 years and is often the first identified feature. However, there is considerable individual variability as to the sequence and combination of clinical features [1]. In addition to the core features of the syndrome, there are a number of other symptoms that have been reported in a range of patients, including metabolic and behavioural symptoms [1,8] (Figure 1); however, the prevalence of these additional features warrants further exploration.

### 4.1. Rapid-Onset Obesity

**Figure 2:**
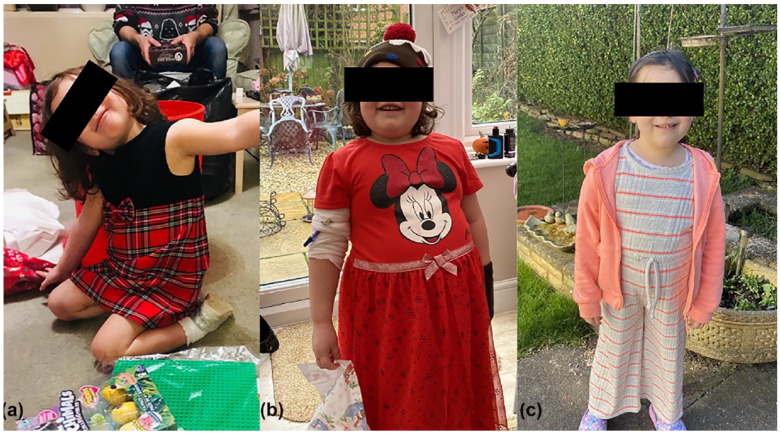
Patient photographs demonstrating the rapid onset of obesity and following treatment with Rituximab. (**a**) Before onset of symptoms; (**b**) 6 months later; (**c**) 12 months after treatment with Rituximab.

Rapid onset of obesity is usually the first identified feature, in a previously well child [4,8], associated with hyperphagia. The median age of onset of weight gain reported from a case series of 43 patients was 3.1 years [1]. This weight gain is often extreme, for instance, 6–10 kg in 6–12 months [12]; however, the actual extent of weight gain is often difficult to quantify due to the lack of routine childhood weight surveillance. Photographic evidence provided by families may support identification of the degree of weight gain (Figure 2). Patients may be misdiagnosed initially with nutritional obesity or presumed to have other syndromes associated with early onset of obesity, such as Prader–Willi syndrome [4,7,16], Bardet–Beidl [5], Smith–Magenis syndrome [18], or HIDEA syndrome [13].

### 4.2. Hypoventilation

Respiratory manifestations of the syndrome include obstructive sleep apnoea (OSA) and nocturnal central hypoventilation [53]. OSA is characterised by prolonged partial upper airway obstruction and/or intermittent complete obstruction that disrupts normal ventilation during sleep and normal sleep patterns [54]. The risk of OSA is increased in children living with obesity in general, with the prevalence of OSA estimated to be between 13 and 59% [55]. The prevalence of OSA in ROHHAD syndrome is estimated to be between 33 and 35% [1,4]. However, in this condition, children may present initially with OSA, which then progresses to central hypoventilation, and therefore ongoing surveillance is required, utilising polysomnography or cardiorespiratory sleep studies, in order to detect central hypoventilation [53,56,57]. In a case series of 28 patients, it was reported that 83% developed central hypoventilation within five years of the onset of obesity [13]. 

In addition to nocturnal respiratory difficulties, some patients develop diurnal hypoventilation requiring daytime ventilation [53]. Respiratory arrest due to central hypoventilation [4,5,35] is the leading cause of death in ROHHAD syndrome [8,21]. There is currently no consensus as to the frequency of sleep studies and evaluation of ventilation when awake, or during exercise, in suspected cases of the syndrome [53]. 

### 4.3. Hypothalamic Dysfunction

Apart from the rapid onset of obesity with hyperphagia, hypothalamic dysfunction in ROHHAD syndrome may involve any combination of growth hormone deficiency (GHD), arginine vasopressin (AVP) deficiency (previously known as central diabetes insipidus), central precocious puberty, hypogonadotropic hypogonadism, hyperprolactinaemia, central hypothyroidism, and adrenocorticotropic hormone (ACTH) deficiency [4,8]. These features commonly develop between 4 and 8 years of age [1]. 

Hypernatraemia is a common early feature secondary to possible hypothalamic dysfunction, with case series reporting prevalence in up to 97% [6], and further investigations may confirm AVP deficiency [1]. Growth hormone deficiency has been reported to occur in approximately 25% of patients [6]. Hyperprolactinaemia is a commonly reported early feature [58], which may be transient, reported in 96% of a cohort of 28 patients [6]. 

As many of these hypothalamic-pituitary endocrine disorders are seen in other conditions or in isolation, this may contribute to misdiagnosis of ROHHAD syndrome until other features present [5]. 

### 4.4. Autonomic Dysregulation

Autonomic disturbances are common in ROHHAD syndrome, often presenting with vague and highly variable symptoms [59] and reported to emerge between 3 and 11 years of age [1]. Different types of autonomic dysregulation encountered include constipation, strabismus, ptosis, cold peripheries, thermal dysregulation, hypothermia, excess sweating, bradycardia, syncopal episodes, and altered pain perception [1,8]. The prevalence of these autonomic features is unknown. Surveillance for autonomic features is beneficial, such as ECG, 24 h Holter monitoring, and serial measures of temperature, because of the wide-ranging symptomatology. 

### 4.5. Neuro-Endocrine Tumours (NETs)

Neuro-endocrine tumours (NETs) are a common, late feature in up to half of cases [60]. Across case reports of 48 patients, 52% developed NETs [6], the most common of which being ganglioneuroma and ganglioneuroblastomas [52,61], with one reported case of a hamartoma mass with neural elements. The median reported age of occurrence of NETs in one case series of 43 patients was 4.75 years (range 4–8.45 years) [1]. ROHHAD has also been described as a paraneoplastic syndrome resulting from NETs [62], although the presence or detection of NETs is not universal in ROHHAD. The majority of NETs in ROHHAD are benign; however, malignant tumours have been reported [63]. Whole body surveillance, such as with magnetic resonance imaging (MRI), is warranted as NETs have been detected in unusual locations, such as in the pelvis [64]. 

### 4.6. Metabolic Features

Metabolic dysfunction is seen in some patients with ROHHAD, which may either be related to the condition itself or be seen as a consequence of obesity. This includes insulin resistance, impaired glucose tolerance, diabetes mellitus, hypertriglyceridaemia, and metabolic dysfunction-associated steatotic liver disease (MASLD) [8,58,65]. 

Dysglycaemia has been reported in a number of individuals, including some children as young as 10 years old developing insulin resistance [65] and type 2 diabetes mellitus (T2DM) [66]. The mechanism of glucose dysregulation is unclear, and as hypothalamic and autonomic dysfunction are key aspects of the condition, dysglycaemia may be related to a central mechanism [58]. 

Hypercholesterolemia, more specifically raised low-density lipoprotein (LDL) and hypertriglyceridaemia, has been reported in some cases from a very young age [1,66]. MASLD, previously known as non-alcoholic fatty liver disease (NAFLD), has been identified in individuals with ROHHAD syndrome as young as four years old [65,66]. The pathophysiology of these metabolic features, often presenting at a young age, has not yet been established.

### 4.7. Neuro-Psychiatric and Behavioural Features

Although not forming part of the diagnostic criteria, a range of behavioural and psychiatric features have been reported in many patients with ROHHAD syndrome. One case series of 15 patients reported that 53% had behavioural symptoms, with 13% having depression [4]. Behavioural changes described include irritability, aggression, hyperactivity, intellectual disability, and decline in school performance, whilst neuro-psychiatric disorders include depression, anxiety, obsessive compulsive disorder, and psychosis [4,5,6,8,15,67,68,69]. There are reports of dramatic behavioural change [49] being the presenting feature of the syndrome. These neuro-psychiatric and behavioural aspects of the condition may not be detected without careful screening and assessment [12], such as through the use of validated questionnaires and neuro-cognitive assessment.

### 4.8. Other Features

There have been various other features reported to be associated with ROHHAD syndrome, such as developmental regression in previously well children [1,14] beginning at 2–3 years of age. Some patients have been described to have seizures [1,6], either generalised tonic–clonic seizures at the time of diagnosis or in association with hypoxaemia [4]. There are also reports of hypersomnolence in patients with the syndrome [1,4], which may possibly relate to poor sleep patterns due to the obstructive sleep apnoea. The prevalence of these features is unknown, particularly as they are not included in diagnostic criteria [4] of this heterogeneous condition. 

## 5. Diagnosis

**Figure 3:**
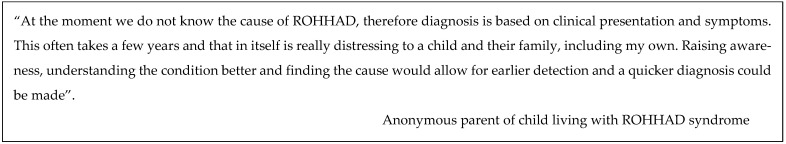
Parent view on diagnosis.

With no specific diagnostic biomarker, as with many rare diseases [70], diagnosis is often challenging and delayed [1]. A lack of awareness within the medical community, further compounded by the fact that individuals with the syndrome may present to a range of different medical specialties, may also contribute to a protracted diagnostic pathway [15]. This may lead to delay in diagnosis [1], difficulties in managing uncertainty for affected families (Figure 3) and poorer clinical outcomes. 

**Figure 4:**
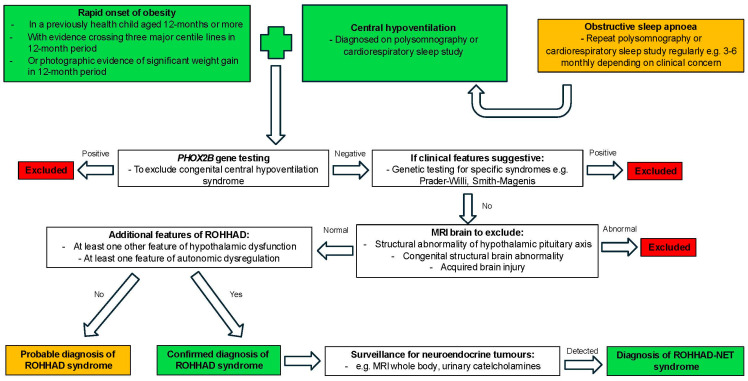
Diagnostic algorithm for ROHHAD syndrome.

Ize-Ludlow et al. proposed criteria for the diagnosis of ROHHAD syndrome when naming the condition in 2007, which included the presence of alveolar hypoventilation after the age of 2 years and evidence of hypothalamic dysfunction as defined by more than one of the following: rapid-onset obesity, hyperprolactinaemia, central hypothyroidism, disordered water balance, failed growth hormone stimulation, corticotropin deficiency, or delayed/precocious puberty [4]. The possibility of a checklist to be utilised in children with rapid-onset obesity and symptoms suggestive of the syndrome has been proposed in order to facilitate earlier diagnosis [12]. A diagnostic algorithm is shown in Figure 4. 

The diagnosis is clinical and involves the exclusion of other conditions with a genetic basis [21]. CCHS should be excluded by testing for *PHOX2B* variants, which are present in approximately 90–100% of those cases [71]. Additionally, it may be beneficial to undertake genetic testing to exclude PWS [8] because the clinical presentation has a number of similarities. Baseline magnetic resonance imaging (MRI) is recommended, regardless of age, to ensure that there are no structural anomalies, which would be suggestive of an alternative diagnosis to ROHHAD syndrome.

Diagnosis is often highly challenging and protracted, being based on a constellation of clinic features that present in different combinations with a variable timeline [1]. The development of diagnostic tests would improve this process for families living with the condition and should be a research priority. 

## 6. Management

Due to its rarity, there has been no systematic approach to treatment [62], and there is no current guideline or consensus statement regarding management. Treatment is largely focused on symptomatic management for each of the clinical features (Table 1). A multidisciplinary approach is required, involving a range of specialisms, including endocrinology, respiratory medicine, dietetics, psychology, sleep physiology, cardiology, neurology, radiology, and often oncology. Consideration is warranted as to how care is delivered in ROHHAD syndrome and the potential benefit of multi-disciplinary, specialist clinics with centralisation of care. There is evidence to suggest that such co-ordination can improve care delivery and reduce the burden on patients and families [72,73]. 

### 6.1. Weight Management

It is important that patients have access to a dietitian and paediatric endocrinologist with expertise in weight management [7], as obesity is likely to contribute to and exacerbate other aspects of the condition. However, little evidence exists regarding successful treatments for hyperphagia, and obesity encountered as part of the condition [74]. Weight management may be targeted at losing weight or maintenance of weight with a corresponding reduction in body mass index (BMI) as the patient grows. 

There is accumulating evidence demonstrating the effectiveness of anti-obesity medications in adolescents, such as glucagon-like peptide 1 (GLP-1) receptor agonists [75]. There has been a report published of one child losing weight, with an improvement in hyperphagia, with semaglutide (GLP-1 receptor agonist) in addition to phentermine [74]. An adult with ROHHAD since childhood treated with liraglutide (GLP-1 receptor agonist) experienced a dramatic reduction in insulin requirements but limited weight loss [65]. Another individual with childhood-onset ROHHAD was treated with semaglutide at 24 years of age and experienced 8.5% weight loss after 12 months of treatment [76]. One patient was described to have lost a significant amount of weight following treatment with rituximab (Figure 2), a monoclonal antibody, along with an improvement in their inflammatory immune profile [35]. Multi-centre research studies would be of value to ascertain the effectiveness of different weight loss interventions [65,74]. 

### 6.2. Respiratory Management

Central hypoventilation, which may be insidious in onset [7] and proceeded by obstructive hypoventilation [56], is a key component of ROHHAD syndrome [4]. Therefore, it is essential that patients with other hallmark features, such as rapid-onset of obesity and hypothalamic dysfunction, should have careful evaluation for hypoventilation utilising polysomnography or a cardio-respiratory sleep study. The respiratory insufficiency seen in this syndrome poses a risk of sudden death and therefore careful surveillance for, and management of, hypoventilation is required [56,77]. 

Patients seem to be particularly susceptible to hypoxaemia in comparison to children with CCHS who are more prone to hypercarbia [53,77]. It has been reported that many patients with ROHHAD syndrome have deficits in awake control-of-breathing, in addition to hypoventilation during sleep [56,77]. Therefore, it is important for patients to have breathing evaluated during wakefulness, as well as sleep [7,53,56,58,59]. It has been suggested that early intervention with nocturnal artificial ventilation may improve daytime ventilation [3]. 

Many patients require bi-level positive airway pressure (BiPAP); however, there is often a progression towards the need for 24 h artificial ventilation, hence requiring a tracheostomy [1,7] in up to 50% of patients [69]. Diaphragmatic pacing has been used in some children for ventilatory support whilst awake [78,79]. Multi-centred studies to explore respiratory deficits both at rest and during physical activity in patients would be beneficial to inform future interventions [53].

### 6.3. Hypothalamic Features

The condition involves a range of hypothalamic features and endocrinopathies and therefore requires specialist endocrine assessment and long-term follow-up. Hyperprolactinaemia is common and often present at diagnosis, possibly transiently [1,6], which may require treatment, for example, with the dopamine D2 receptor agonist cabergoline [58]. Patients may present with, or develop, growth hormone (GH) deficiency. Thus, poor linear growth and height velocity require appropriate investigation with growth hormone stimulation tests to assess growth hormone secretion and replacement if deficiency is identified. There is a recognised risk that GH replacement may impact airway obstruction in patients with PWS and sleep apnoea [80], posing a theoretical risk in ROHHAD syndrome [5]. 

Disorders of sodium and water regulation are common, including Arginine vasopressin deficiency (AVD) and transient syndrome of inappropriate antidiuretic hormone (SIADH) [1,21]. Thus, symptoms of polyuria and polydipsia should be addressed and investigated if present because patients may require treatment with desmopressin (DDAVP) and fluid intake monitoring in the case of AVD. Patients may develop central hypothyroidism, so monitoring thyroid function, including both free T4 and TSH levels, is mandatory. ACTH deficiency can sometimes occur, and it is important that patients are tested for this, for example, through a synacthen test, and given appropriate steroid replacement if required. 

Disorders or puberty, including central precocious puberty and hypogonadotropic hypogonadism, may be encountered [5]. These require careful history, pubertal staging, and appropriate investigation [65]. Regularly assessing hypothalamic-pituitary function is mandatory, although no agreement on frequency is published. 

### 6.4. Autonomic Dysregulation

Dysautonomia is heterogeneous and often difficult to identify. Temperature dysregulation, consisting of both hyper- and hypothermia, impaired sweating, or Raynaud’s phenomenon, is frequently encountered, and this may be investigated with serial core and peripheral temperature recordings and where possible measurement of ability to sweat, for example, using a Q-SWEAT test or a thermoregulatory sweat test [1,7,10]. Temperature dysregulation is difficult to manage with limited evidence regarding effective treatment, and basic strategies are often utilised, such as varying clothing appropriately and drinking warm fluids. Altered pain perception may also present [1], and management is limited to taking additional safety precautions. 

Gastrointestinal dysmotility, characterised by constipation or chronic diarrhoea, is recognised as a possible autonomic manifestation [4]. In severe cases, chronic diarrhoea has also been reported to be associated with rectal prolapse [4,49] and therefore input from a gastroenterologist may be required. 

Cardiac features, include heart rate variability and arrhythmias [7,78]. It is important that patients are appropriately investigated with ECG and 24 h Holter monitoring. 

Additionally, ophthalmological manifestations have been reported, such as squint, pupillary dysfunction, oculomotor apraxia, and ptosis [4,14], again with limited available interventions. 

It is important to consider the complexities of managing autonomic dysregulation, along with respiratory compromise, during anaesthesia if patients require surgical interventions [81]. If patients with this condition are required to undergo general anaesthesia, it has been proposed that short-acting anaesthetics with minimal respiratory effects, combined with anxiolytics, are the most appropriate [82]. Furthermore, careful pre-operative optimisation [81] and close post-operative follow-up are recommended due to the high risk of complications, with consideration of different aspects of the condition, such as electrolyte abnormalities, seizure disorders, hormone deficiencies, thermal dysregulation, and behavioural disorders [82]. The wide-ranging impact of autonomic symptoms for patients should not be underestimated. 

### 6.5. Neuroendocrine Tumours

Whilst screening for NETs is essential, no consensus has been reached on frequency nor methodology [1]. Investigation for NETs is important at initial diagnosis [6] with imaging such as whole-body MRI, chest X-ray, and/or adrenal ultrasound scan and measurement of urinary catelcholamines and plasma metenephrines [7], and regular surveillance thereafter. Annual screening has been proposed based on other conditions posing a risk of neuro-endocrine tumours, and more frequent screening may be beneficial initially, [1], but needs to be balanced with the risk of anaesthesia if an MRI is required in a young child. 

If NETs are identified, they should be managed with careful consideration of the complexities of anaesthesia in patients with central hypoventilation and autonomic dysregulation [78,81]. Resection of NETs is associated with good long-term outcomes [60]; however, there is no evidence to suggest this effects the long-term progression of other features [7]. Following tumour staging, intensive treatment may be required [1,61,63]. Further characterisation of NETs in ROHHAD through multi-centre studies would enable the development of more informed guidance around screening and management. 

### 6.6. Metabolic Features

The aetiology of metabolic features that may occur is poorly understood and is a management variable. Glucose dysregulation and progression to type 2 diabetes mellitus have been reported, and continuous glucose monitoring (CGM) may be useful in order to characterise early dysglycaemia [58]. Treatment with metformin, insulin or GLP-1 receptor agonists has been used with variable effects on glucose control, with reports of hypoglycaemia [58,65]. The mechanism of glucose dysregulation and diabetes in these patients is unclear, possibly related to the characteristic hypothalamic or autonomic dysfunction or even the profound obesity [83]. 

MASLD may be seen [65,66] but has no proven effective treatment, apart from weight loss. Dyslipidaemia may present at a young age, and there is a report of statin therapy being commenced in a 5-year-old child [66] with limited effectiveness and possible behavioural side effects. Patients are at risk of developing metabolic syndrome, and cardiovascular disease may contribute to the high mortality risk in this condition [8].

### 6.7. Neuro-Psychiatric and Behavioural Features

Behavioural and psychiatric features are variable, and rigorous clinical evaluation is necessary. A multi-professional approach is warranted, with access to clinical psychology and psychiatry input. Anti-psychotics, antidepressants, and methylphenidate have been used in the past [6,68,79], and benzodiazepines have been used with caution for anxiety associated with the condition [79]. Cyclophosphamide, which is an immunoregulating agent, has also been reported to demonstrate a positive effect on behaviour and neuropsychological function [67]. It is unclear whether psychiatric features respond to conventional treatment, and the use of pharmacotherapy needs to be balanced against the risk of respiratory depression and further weight gain in this complex condition [79]. 

### 6.8. Immunotherapies

Immunotherapies have been used to target a possible underlying autoimmune aetiology with variable response. Cyclophosphamide, an alkylating agent, has been reported to have a stabilising effect on weight and behavioural features of the condition in at least three individuals [36,67,69]. It has been suggested to start at a low dose of cyclophosphamide initially (such as 750 mg/m^2^/every 28 days), and if there is no response, this could be increased to high-dose cyclophosphamide (50 mg/kg/day for 4 consecutive days) [36]. However, immunosuppression and associated septic shock were described in one patient with ROHHAD following treatment with cyclophosphamide [69]. Rituximab, a monoclonal antibody, has been reported to be associated with weight loss (Figure 2), an improved cytokine profile, and autonomic features [35,36]. IV immunoglobulins and various steroid preparations have also been used with variable benefit [6]; however, all are limited to case reports. Larger-scale studies to identify effective immunotherapeutic agents and appropriate dosing schedules, with corresponding measurement of a range of parameters, including cytokine profiles, are warranted to establish efficacy.

## 7. Prognosis

ROHHAD syndrome is associated with a significant mortality risk, with a risk of cardiorespiratory arrest (50–60%) [21] and numerous reports of children dying from the condition during early childhood (aged 4–7 years), although this estimate may be subject to reporting bias [1,3,56]. Data on life expectancy and long-term outcomes of patients are lacking [59], with limited reports of patients having reached adulthood [57,69,84]. There is some evidence to suggest that with early recognition, intensive multi-disciplinary management outcomes may be improved [7,59]. Further research is needed to characterise the progression of the syndrome, and how this is modified by intervention to improve understanding of prognosis to communicate with patients and their families, thus improving care [14].

## 8. Conclusions

Despite some research, ROHHAD remains enigmatic in many respects. A crucial unknown aspect is its aetiology. A range of genetic, epigenetic, autoimmune, and neuro-anatomical aetiologies have been speculated and investigated but none proven. This devastating condition merits additional attention in order to enable prompt diagnosis and disease management therapy. 

Diagnosis continues to be difficult and prolonged, particularly without a diagnostic biomarker and lack of awareness of the condition amongst clinicians. However, the emergency of ZSCAN1 antibodies as a potential diagnostic tool is promising and could enable earlier diagnosis. Further research into the specificity and sensitivity of these antibodies and wider access to appropriate assays to test for them would be beneficial. 

Whilst causation is unestablished, the development of targeted treatments is very challenging. Multi-centre studies are needed to develop and evaluate treatments for different aspects of the condition. Once aetiology is known, finding curative treatment targeted at the underlying aetiology must be a research priority. 

In addition, it would be beneficial for clinical recommendations to be developed as to the most appropriate mode and frequency of screening for different clinical features of the condition, including NETs. This could be enabled through international collaborative research utilising all available evidence of the natural history of the syndrome. Similarly, a greater understanding of outcomes, by detailed long-term follow-up of identified cases would enable better information for patients and their families and identify future likely service needs. Although a parentally reported international ROHHAD registry has been established [85], an international data repository with regular clinician reporting would be invaluable.

The impact of the wide-ranging symptoms of the syndrome on an individual and their family, combined with the stress of living with an uncertain prognosis [86], cannot be underestimated. However, studies evaluating the lived experience of families are currently lacking and would be beneficial to inform care provision [87] and research priorities. Collaboration between patient organisations, charities, and the healthcare sector at a multinational level is key to enabling this. Planning service provision for individuals with ROHHAD syndrome is challenging without an accurate estimate of incidence and prevalence, natural history, or clinical outcomes. Longitudinal surveillance studies in order to characterise these are urgently required.

This review provides a comprehensive synthesis of what is currently known regarding this syndrome, with a focus on identifying outstanding areas for research. Limitations of this work include a lack of novel reported findings and the fact that there are so many unanswered questions. 

There remains much to be known about this elusive disease. However, ongoing collaborative research at a multi-national level to progress understanding, and management, of ROHHAD syndrome will enable crucial improvements in patient care and outcomes. 

## Figures and Tables

**Table 1:** Management strategies.

Clinical Feature		Diagnostic Tests	Potential Interventions
Hypothalamic dysfunction	Obesity	Growth chart monitoring Photographs	Dietary modification Physical activity Pharmacotherapy, e.g., GLP-1 RA
	Hyperprolactinaemia	Repeated blood tests	Cabergoline
	Growth hormone deficiency	Growth hormone stimulation test, e.g., ITT	Growth hormone
	Diabetes insipidus	Paired osmolalities, water deprivation test	DDAVP
	Adrenal insufficiency	Synacthen test	Hydrocortisone
	Precocious puberty	Pubertal examination Pelvic USS, bone age X-ray, LHRH test	Triptorelin, e.g., decapeptyl or gonapeptyl
	Hypothyroidism	Thyroid function tests	Thyroxine
Respiratory abnormalities	Obstructive sleep apnoea	Cardiorespiratory sleep studies Polysomnography	CPAP
	Central hypoventilation		BiPAP, tracheostomy
Autonomic dysregulation	Altered sweating	Q-Sweat test Thermoregulatory sweat test	Iontophoresis
	Hypothermia / hyperthermia	Serial measurement of core and peripheral temperatures	Warming or cooling techniques
	Arrythmias	ECG 24 h Holter monitoring	Anti-arrhythmic agents
	Ophthalmological manifestations	Ophthalmological assessment	Prescription glasses
	Constipation	Clinical history and examination	Stool softeners, stimulant laxatives
Neuro-endocrine tumours	Ganglioma	MRI, USS adrenals	Surgical removal Ongoing surveillance
	Ganglioneuroblastoma	Plasma metanephrines, urinary catelcholamines	
Metabolic features	Type 2 diabetes mellitus Impaired glucose tolerance	Fasting glucose and insulin, HbA1c OGTT	Metformin, insulin GLP-1 RA
	Dyslipidaemia	Fasting lipid profile	Statin therapy if required
Behavioural and neuro-psychiatric features	Depression and anxiety	Validated questionnaires, psychiatric assessment Neuro-cognitive assessment	Anti-depressants
	Emotional instability		Behavioural therapies

GLP-1 RA = glucagon-like peptide-1 receptor agonist; ITT = insulin tolerance test; DDAVP = desmopressin; USS = ultrasound scan; LHRH = lutenising hormone releasing hormone; CPAP = continuous positive airway pressure; BiPAP = bilevel positive airway pressure; ECG = echocardiogram; MRI = magnetic resonance imaging, HbA1c = glycated haemoglobin; OGTT = oral glucose tolerance test.

## Data Availability

No new data were created or analysed in this study.
